# Uncertainty‐aware gamma interaction localization and reconstruction in PET

**DOI:** 10.1002/mp.70471

**Published:** 2026-05-14

**Authors:** Julian Thull, Jan Remennik, David Schug, Bjoern Weissler, Yannick Kuhl, Volkmar Schulz

**Affiliations:** ^1^ Department of Physics of Molecular Imaging Systems RWTH Aachen University Aachen Germany; ^2^ Chair of Imaging and Computer Vision RWTH Aachen University Aachen Germany; ^3^ Hyperion Hybrid Imaging Systems GmbH Aachen Germany; ^4^ Fraunhofer Institute for Digital Medicine MEVIS Bremen Germany

**Keywords:** gamma positioning calibration, PET image reconstruction, uncertainty quantification

## Abstract

**Background:**

Precise localization of gamma‐ray interactions inside scintillation detectors is essential for high‐resolution positron emission tomography (PET) imaging. Although machine learning methods have demonstrated strong performance in gamma interaction positioning, most existing approaches do not quantify event‐level uncertainty, leaving valuable information unused.

**Purpose:**

This study quantifies event‐wise positional uncertainties in gamma interaction localization and demonstrates its utility for improving PET image quality through uncertainty‐aware filtering and weighting.

**Methods:**

We employ machine learning to perform gamma interaction positioning in a semi‐monolithic scintillation detector block with overall dimensions of $48.4\times 48.3\times 10\;{\mathrm{mm}}^{3}$, corresponding to the planar‐segmented, planar‐monolithic, and depth‐of‐interaction (DOI) directions. We train multilayer perceptrons (MLPs) and convolutional neural networks (CNNs) using silicon photomultiplier (SiPM) light spread measurements as network inputs. Regression models were trained using a Gaussian negative log‐likelihood to jointly estimate gamma interaction coordinates and event‐wise positional variance. Classifiers were evaluated, which inferred event‐level uncertainty from the variance of the predicted spatial probability distribution. These uncertainty estimates primarily reflect variability in detector response and photon statistics, corresponding to aleatoric uncertainty. Regression and classification objectives were explored through task‐specific hyperparameter optimizations. All 24 semi‐monolithic detectors of a proof‐of‐concept PET system were calibrated in the segmented, monolithic, and depth‐of‐interaction (DOI) dimensions using collimated fan‐beam irradiations of each detector. For each detector, a dataset of 880,000 events per spatial dimension was acquired and randomly split into 60% training, 20% validation, and 20% test sets, with models trained and evaluated independently across seven detectors. The resulting calibration models were then applied to reprocess measurements of imaging phantoms. Performance was evaluated using the mean absolute error (MAE) on the fan‐beam dataset and the median distance between reconstructed lines of response (LORs) and known point‐source locations measured within the scanner. Predicted variances were further integrated into the time‐of‐flight ordered‐subsets expectation maximization (TOF‐OSEM) reconstruction via event‐level filtering and LOR weighting to assess spatial resolution and noise propagation.

**Results:**

Across architectures, uncertainty‐aware models achieved high positioning accuracy. CNN classifiers provided the best planar performance, while CNN regressors performed best for depth‐of‐interaction (DOI) estimation. Variance‐ and energy‐based filtering substantially improved positioning accuracy, reducing the MAE to 0.47mm in the monolithic and 0.83mm in the DOI dimension. Variance‐ and energy‐aware filtering also improved the LOR precision, reducing the median LOR distance to as low as 0.98mm. In image reconstruction, filtering and weighting improved image quality, with filtering providing the strongest gains and enabling visualization of 0.8mm rods with a peak‐to‐valley ratio (PVR) of 1.184. These approaches increased the signal‐to‐noise ratio (SNR) but also the coefficient of variation (COV), consistent with reduced effective sensitivity and amplified Poisson noise.

**Conclusions:**

Event‐level uncertainty estimation enables meaningful filtering and weighting strategies that improve interaction positioning and reconstructed PET image quality. Despite the trade‐off between enhanced spatial resolution and increased noise, the uncertainty‐aware framework introduces a new reconstruction parameter that can be exploited to improve image quality and potentially support more reliable quantitative PET imaging.

## 1 INTRODUCTION

high‐resolution Positron Emission Tomography (PET) imaging fundamentally depends on the precise characterization of individual gamma‐ray interactions inside scintillation detectors. Besides precise timing and energy estimation, accurate interaction localization is crucial, as it determines the precision of the resulting lines of response (LORs) and, consequently, the achievable spatial image resolution. Clinically, improvements in spatial resolution translate into enhanced visualization of small or low‐contrast lesions and more accurate tumor segmentation and therapy response assessment.^1^, ^2^, ^3^ In PET, the inferred positions of 511keV photon interactions define the lines of response (LORs) along which annihilation photon pairs are assumed to propagate. These photons, produced by a $\beta^{+}$ decay followed by positron–electron annihilation, travel in near‐opposite directions due to momentum conservation before reaching scintillation crystals coupled to silicon photomultipliers (SiPMs). Precise determination of these interaction points ensures precise LOR placement and, consequently, the correct intersection with the underlying radioactive tracer distribution within the patient. Nonetheless, reliable estimation of interaction positions remains challenging. Crystal nonuniformities, electronic noise in the detector readout, and the stochastic nature of gamma‐matter interactions affect the observed scintillation light distributions, introducing uncertainty in reconstructed interaction positions and ultimately limiting achievable image resolution and diagnostic performance. This work addresses these challenges by modelling event‐level positional uncertainty, defined as the event‐dependent expected deviation between the true and estimated interaction position, and by evaluating its impact on system‐level PET performance using a real PET scanner based on the semi‐monolithic detector design.^4^

The photoelectric effect leads to a full absorption of the incident 511keV gamma photon energy and produces a localized scintillation response. In typical PET scintillators like lutetium–yttrium oxyorthosilicate (LYSO), however, Compton scattering dominates at this energy,^5^, ^6^ leading to partial energy transfer, and continued transport of scattered photons within the detector. Each interaction generates a scintillation pulse that propagates through the crystal and produces a characteristic response in the coupled SiPM array. When multiple interactions occur, their corresponding light spreads superimpose, producing composite patterns that impair precise localization of the initial interaction. Additional distortions arise from reflections at crystal boundaries, spatially varying light transport due to material imperfections, and nonideal optical coupling between the scintillator and SiPM array.^7^, ^8^ Electronic noise sources, including SiPM dark counts, temperature‐ and bias‐dependent gain variations, baseline noise, and timing jitter introduced by signal amplification and digitization, additionally influence the measured light signals in an event‐dependent nonlinear manner.^9^, ^10^ Despite their relevance, the uncertainty introduced by such noise sources is rarely quantified in existing PET reconstruction pipelines, leaving potential gains in image quality largely unexplored.

Due to the aforementioned effects influencing scintillation signal formation, conventional analytical methods, such as the center‐of‐gravity (COG), face substantial limitations. Neither multiple light clusters nor reflections at crystal boundaries are handled well by this approach, substantially restricting its applicability for modern (semi‐)monolithic detector blocks. In light of these limitations, machine learning techniques have gained increasing attention as data‐driven alternatives capable of capturing these nonlinear effects. Gradient‐boosted trees (GTB) have demonstrated strong performance as calibration backbone models for gamma positioning, improving both planar and depth‐of‐interaction (DOI) accuracy, while also being well suited for direct FPGA deployment.^11^, ^12^, ^13^ Neural‐network‐based approaches have likewise been explored,^14^, ^15^, ^16^, ^17^, ^18^, ^19^, ^20^ including multilayer perceptrons (MLP), convolutional neural networks (CNN), and architectures incorporating residual connections. Similar to GTBs, these models leverage their capacity to learn complex nonlinear mappings between scintillation light distributions and interaction coordinates, thereby improving both planar as well as DOI positioning. To address the problem of inter‐crystal scatter in particular, Lee et al.^21^ used two dedicated CNNs. The first network (ICS‐eNet) estimated the energy distribution across the crystal array to identify scattered events, which was then used to identify inter‐crystal scattered events and interacted crystals. The second (ICS‐cNet) network predicted the first interaction point from raw sensor signals. Both methods yielded notable improvements in spatial resolution. To improve positioning performance in pixelated PET detectors with light‐sharing readout, several groups have also investigated maximum‐likelihood (ML)‐based methods. Notably, the work by Gross‐Weege et al.^22^ showed that ML estimation offers greater robustness to missing photodetector signals than the conventional COG method. Lerche et al.^23^ proposed an alternative framework that jointly estimates both the interaction pixel and deposited energy using an iterative formulation, resulting in improved energy resolution and positioning performance. More recently, the group introduced an optimized, non‐iterative variant designed to meet the processing requirements of modern high‐count‐rate PET systems.^24^ To address positional uncertainty in calibration models, Daniel et al.^25^ proposed a fully connected neural network trained with a truncated Gaussian negative log‐likelihood (NLL) loss to jointly estimate interaction position and associated uncertainty, expressed as the variance of the truncated Gaussian. However, the study is limited to simulation‐based detector‐level evaluations and does not investigate how such event‐level uncertainty estimates propagate through calibration and impact reconstructed PET image quality. Nonetheless, the work provides an important theoretical indication that incorporating interaction‐level uncertainty estimates has the potential to enhance PET imaging performance.

This work extends prior studies on uncertainty quantification in PET gamma‐interaction localization through a system‐level evaluation conducted on a proof‐of‐concept PET scanner featuring 24 semi‐monolithic detector blocks. Deep neural network architectures are optimized for interaction positioning using a heteroscedastic loss formulation that jointly predicts the first gamma interaction position and variance estimates as an uncertainty proxy, supported by large‐scale hyperparameter optimization using the Optuna framework.^26^ Fully connected and convolutional neural networks are evaluated in regression and classification settings. The networks are trained using a collimated fan‐beam dataset, which provides labels corresponding to the first gamma interaction position, such that inter‐crystal scattering and multiple interaction events are implicitly learned in a probabilistic manner during training. We further investigate event filtering and weighting strategies that incorporate predicted variances into image reconstruction. After describing the detector, scanner, and calibration setup, we detail the training procedure and evaluation methodology. Positioning performance is then assessed on both the collimated fan‐beam dataset and a dataset of measured point sources inside the scanner, complemented by an analysis of the relationship between predicted variance, deposited energy, and localization error, followed by quantitative comparisons of reconstructed images. Finally, we discuss implications of these findings, outline potential applications and challenges, and identify directions for future research.

## 2 MATERIALS AND METHODS

### 2.1 Semi‐monolithic slab detector scanner

**FIGURE 1:**
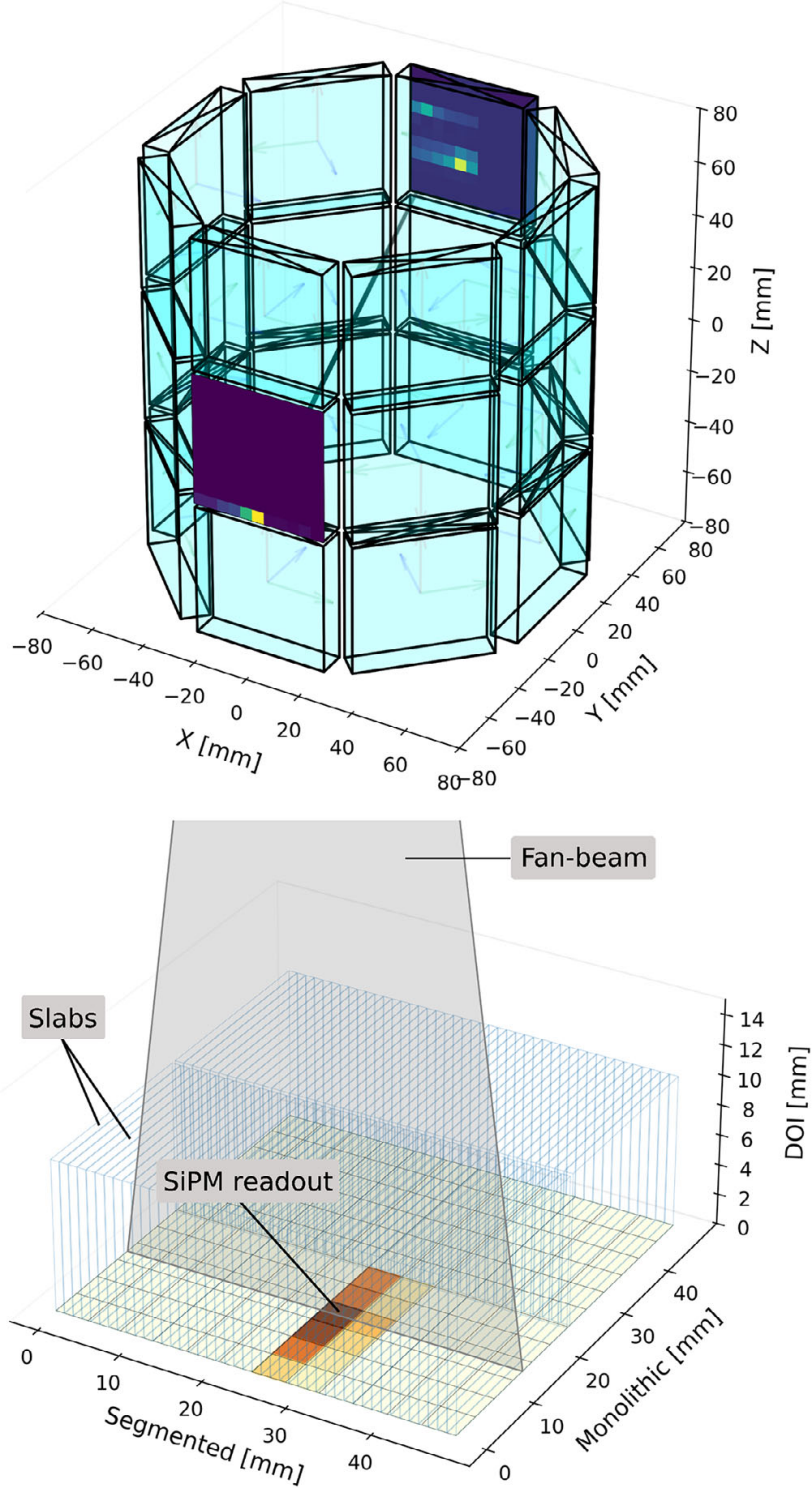
(Top) Detector arrangement of the proof‐of‐concept scanner illustrating an example LOR and corresponding scintillation light distributions of both gamma interactions. One gamma photon undergoes Compton scattering within the detector volume, generating two spatially separated light clusters, thereby introducing ambiguity in identifying the first interaction. (Bottom) Schematic of the semi‐monolithic detector block with a fan‐beam configured for monolithic irradiation, adapted from Thull et al.^27^ The detector has overall dimensions of $48.4\times 48.3\times 10\;{\mathrm{mm}}^{3}$, corresponding to the planar‐segmented, planar‐monolithic, and DOI directions, respectively.

We utilize the finely segmented semi‐monolithic LYSO scintillation detector.^4^ The detector block is made of two slab arrays, each consisting of 44 polished LYSO slabs of dimensions $1\times 24\times 10\;{\mathrm{mm}}^{3}$, separated and surrounded by a 0.1mm reflective ${\mathrm{BaSO}}_{4}$ layer. The slab arrays are covered by a retroreflector on the top surface and wrapped in aluminum foil on the outer surfaces for optical isolation. A 0.3mm light guide separates the crystal array from the 12×12 SiPM array, implemented using a 6×6 grid of Philips DPC3200‐22 sensors, each with 2×2 digital channels,^28^ mounted on the Hyperion DPC‐Tile board.^29^ The detector is visualized in Figure 1. This semi‐monolithic geometry enables precise planar interaction localization and DOI estimation by exploiting variations in the scintillation light spread along the monolithic axis. Specifically, interactions close to the photosensor generate focused light distributions on the sensor array, whereas interactions occurring farther from the photosensor produce wider and more diffuse light patterns. These depth‐dependent signal patterns can be calibrated and used for DOI estimation. To fully exploit the detector's potential, robust positioning calibration for all three spatial dimensions is required, as presented in the following sections. Even though the segmented direction would in principle allow for analytical positioning models based on simplified light‐sharing assumptions, the actual detector response exhibits nonlinearities due to optical boundary effects, inter‐slab light‐sharing, and sensor non‐idealities. In particular, edge regions and multi‐interaction events lead to asymmetric and overlapping light distributions that are not well captured analytically, making a data‐driven approach more robust and statistically efficient. We therefore employ machine learning for all three spatial dimensions. Energy calibration was performed using a position‐dependent approach.^30^ For this purpose, the detector volume was virtually discretized into 44×48×4 voxels, representing virtual calibration bins rather than physical segmentation, with voxel sizes chosen to ensure sufficient statistics per bin. Computed energies contain the deposited energy across the whole detector, including all interaction clusters. The achieved energy resolution is approximately 12.7%. For timing, a time‐skew correction was applied to each detector block, achieving a coincidence timing resolution (CTR) of approximately 500ps. For a comprehensive in‐depth description of the detector and applied calibration procedures, refer to Kuhl et al.^4^

A total of 24 semi‐monolithic detector blocks were manufactured and arranged in a cylindrical geometry at an approximate inner radius of 62.5mm, cf. Figure 1. System control was implemented using the Hyperion III PET and PET/MRI platform.^31^ This enabled the collection of raw sensor data for offline analysis and reprocessing of acquired measurements using updated calibration models.

### 2.2 Benchtop‐calibration dataset

A labelled dataset for positioning calibration was acquired using a collimated fan‐beam setup with a fan width of 0.5mm and a motorized stage with a positioning repeatability of 2μm.^4^ Light spreads were recorded and paired with the known positions of the fan‐beam irradiation positions along the segmented, monolithic, and DOI dimensions. Data collection involved irradiating the detector in defined orientations:

- along the segmented axis (44 positions with a 1.1 mm pitch and 20,000 collected samples per target),
- perpendicular to the slabs for monolithic calibration (96 positions with a 0.5 mm pitch and 9,166 collected samples per target),
- and parallel to the photosensor for DOI calibration (40 positions with a 0.25 mm pitch and 22,000 collected samples per target).

A separate labelled dataset was acquired per detector and per calibration dimension, where the eight detectors located in the scanner's central ring provide labels for all three dimensions (with one exception lacking segmented‐axis labelling), whereas the sixteen detectors in the two outer rings provide labels only for the monolithic and DOI axes. For the segmented direction, detector‐specific models were trained for the central ring detectors, while the outer ring detectors used a model trained on a central ring detector, as this dimension is primarily determined by the crystal slab geometry and exhibits low inter‐detector variability. A single collected sample corresponds to one detector readout represented by its scintillation light distribution on the photosensor array, which may include multiple interaction clusters. The event energy was reconstructed from the same readout using an energy calibration model. No energy window was applied during data acquisition; instead, only readouts with more than 500 detected photons were retained for noise reduction and signal quality, while explicit energy windowing was applied later during evaluation. Although detector‐specific benchtop calibration is time‐consuming and labor‐intensive, the proposed method is independent of the specific calibration data acquisition procedure. Recent advances in in‐system calibration indicate that comparable datasets can be collected via virtual collimation, reducing data acquisition times to hours and substantially lowering manual labor overhead.^32^ The complete benchtop calibration dataset for all detectors used in this work was acquired over several weeks. The dataset was split into training 60%, validation 20%, and test 20% sets.

### 2.3 Neural network models

Multilayer perceptrons (MLPs) and convolutional neural networks (CNNs) were employed as calibration backbone models for both classification and regression. Using the fan‐beam dataset, separate models were trained for each spatial dimension (segmented, monolithic, DOI) for each detector block. For detectors where segmented calibration data were unavailable, the segmented model from a central ring detector was used instead. In total, 55 calibration networks were trained across all detectors and spatial dimensions (7 central detectors × 3 models, 1 central detector × 2 models, and 16 outer detectors × 2 models). Each model received 144 features corresponding to the photon counts from the 12×12 SiPM array, with input dropout simulating missing readout channels. The MLP architecture comprized stacked fully connected layers with batch normalization (BN), rectified linear unit (ReLU) activation, and dropout. The output layer produced either softmax class probabilities for classification or two sigmoid‐scaled regression outputs for interaction coordinate and associated uncertainty. For CNNs, convolution–BN–ReLU–dropout blocks were used to extract spatial features, followed by a fully connected module analogous to the MLP design. The final layer similarly yielded either softmax probabilities or sigmoid‐scaled coordinate and uncertainty estimate.

For regression, each model directly predicts the interaction position as a scalar mean ${\hat{y}}_{n}$, along with an associated uncertainty $\sigma_{n}^{2}$, which we optimize using the Gaussian NLL loss:

(1) $$L_{\text{NLL}}=\sum_{n=1}^{N}\left[\frac{1}{2}\log\left(\sigma_{n}^{2}\right)+\frac{{\left(y_{n}-{\hat{y}}_{n}\right)}^{2}}{2\sigma_{n}^{2}}\right]$$

where $y_{n}$ is the ground truth position for sample n, and N is the number of samples in the minibatch. For classification across C positional bins, models are trained using the categorical cross‐entropy loss:

(2) $$L_{\text{CE}}=-\sum_{n=1}^{N}\sum_{c=1}^{C}y_{n,c}\log\left({\hat{p}}_{n,c}\right)$$

where $y_{n,c}$ is the one‐hot‐encoded ground truth and ${\hat{p}}_{n,c}$ is the softmax‐normalized predicted probability for class c and sample n. This distribution defines a discrete probability mass function over position bins. The most probable class is used as the positional estimate, while the uncertainty is quantified as the variance of the predicted distribution.

### 2.4 Hyperparameter optimization (HPO)

HPO for all MLP and CNN models was performed using the Optuna framework,^26^ and applied to one representative semi‐monolithic slab detector. The representative detector was selected randomly, as all detector blocks were manufactured identically and exhibited comparable optical and electronic response characteristics, allowing the optimized hyperparameters to be transferred to the remaining detectors. In total, 12 independent HPO runs were conducted, covering all combinations of network type (MLP/CNN), prediction task (regression/classification), and calibration dimension (segmented/monolithic/DOI). For each configuration, an average of 377 trials were evaluated. Models were trained for up to 50 epochs and evaluated on a held‐out validation dataset, with early termination of underperforming trials using a median pruner and tree‐structured Parzen estimator (TPE)–based sampling of new hyperparameter configurations.^33^ Optuna varied the learning rate, input dropout rate, MLP depth and width, CNN architectural parameters including the number of convolutional blocks, kernel sizes, and feature map dimensions, as well as the size of the final fully connected layer. Early stopping was employed, halting training after 20 consecutive epochs without improvement of the validation loss. Final detector calibration models were trained using the selected configurations for up to 300 epochs using the same early stopping criterion.

### 2.5 List‐mode processing

The final detector calibration models were applied to raw SiPM data acquired during phantom measurements to generate list‐mode datasets suitable for image reconstruction. All detector‐specific models were loaded into VRAM simultaneously, and the singles were routed through their corresponding models using index‐based masking. This enabled highly efficient batched inference within our PyTorch^34^ implementation. The list‐mode format was extended to include per‐interaction variance estimates for both gamma events in all three spatial dimensions. These additional fields enabled uncertainty‐aware algorithms during the subsequent image reconstruction. Data from three acquisitions were processed: a cylindrical shell phantom, a HotRod image‐quality phantom, and a point‐source dataset. The cylindrical shell phantom and the HotRod phantom were filled with 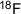
‐FDG, with the latter containing rods of diameters 2, 1.5, 1.2, 1, 0.9, and 0.8mm. The shell phantom was used to derive scanner normalization (i.e., the sensitivity map), while the HotRod phantom was used to assess spatial resolution and image uniformity. The initial activity of the HotRod scan was approximately 5MBq.

In addition, a point‐source dataset was acquired using a 1.5MBq 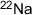 source, mounted on a 3D linear motor stage and systematically translated to randomly selected locations at radial distances of 25 mm and 35 mm from the scanner center. An average acquisition time of 3.1s per position was used, resulting in approximately 491,000 coincidence events per position on average. No data were recorded while the source was moving between positions, as the motor stage and PET acquisition software were synchronized. The dataset was used to evaluate LOR precision.

### 2.6 Image reconstruction

We reconstructed images using the ordered subsets expectation maximization (OSEM) algorithm^35^ from list‐mode time‐of‐flight (TOF) data, processed with the different calibration models. We incorporated the predicted positional uncertainties through event‐level filtering as well as LOR weighting. Uncertainty per detected gamma interaction was quantified using the norm of the predicted variances:

(3) $$\begin{array}{c} \sigma_{\text{3D}}=\sqrt{\sigma_{\text{seg}}^{2}+\sigma_{\text{mono}}^{2}+\sigma_{\text{doi}}^{2}}. \end{array}$$

Filtering was applied at the singles level: a coincidence was accepted only if neither interaction exceeded a threshold $\sigma_{\text{thr}}$. For weighting, we computed an uncertainty score for each LOR as the product of the two single‐event uncertainties of interactions $I_{1},I_{2}$ and converted this measure into a reconstruction weight using:

(4) $$\begin{array}{cc} w_{\text{LOR}} & =\frac{1}{{\left(\sigma_{\text{3D},I_{1}}\cdot \sigma_{\text{3D},I_{2}}\right)}^{p}}, \end{array}$$

where the exponent p controls the strength of uncertainty suppression during OSEM reconstruction. Detection efficiencies were estimated during normalization using the fan‐sum method,^36^ where for crystal i with associated events $K_{i}$ the ratio of the recorded counts $C_{i}$, and the tracer coverage of the possible LORs of the crystal $T_{i}$, defines the detection efficiency $E_{i}=C_{i}/T_{i}$. Count histogramming was performed for all recorded events, the filtered events only, as well as for the variance‐weighted events, where 1[·] denotes the indicator function:

(5) $$\begin{array}{cc} C_{i}^{\mathrm{filt}} & =\sum_{k\in K_{i}}1\;\left[\sigma_{\text{3D},k}\leq \sigma_{\mathrm{th}}\right], \end{array}$$

(6) $$\begin{array}{cc} C_{i}^{\mathrm{wgt}} & =\sum_{k\in K_{i}}\frac{1}{\sigma_{3D,k}^{p}}. \end{array}$$

The tracer coverage within the phantom was obtained by computing line integrals over the detector coincidence matrix. The pairwise product of two crystal efficiencies defined LOR‐specific detection efficiencies, which were backprojected over all possible LORs to generate the sensitivity map. Image reconstruction was performed on a list‐mode basis, enabling both filtered and $w_{\text{LOR}}$‐weighted backprojection of LORs. Because filtering and LOR weighting modify the effective detection sensitivity, quantitative accuracy requires consistent normalization. Since sensitivity estimation is performed under the same filtering and weighting conditions, accepted or weighted counts are scaled consistently to the corresponding tracer coverage, preserving quantitative accuracy while primarily affecting the noise–resolution trade‐off rather than introducing systematic bias. Reconstruction was applied to the processed HotRod image quality phantom measurement. We uniformly sampled 20 LORs per coincidence, constrained to the crystal volume boundaries of both interactions. We then performed 20 iterations with 10 subsets and an image voxel size of 0.2×0.2× $0.2\;{\mathrm{mm}}^{3}$. Unless stated otherwise, a total of 250 million coincidences were used for reconstruction. All reconstructions employed an energy window of 511keV ± 80keV.

### 2.7 Evaluation metrics

We evaluated model performance using the per‐target mean absolute error (MAE) and positioning bias on the test split of the fan‐beam dataset:

(7) $$\mathrm{MAE}\left(t\right)=\frac{1}{N_{t}}\sum_{i\in I_{t}}\left|y_{i}-{\hat{y}}_{i}\right|,$$

(8) $$\mathrm{Bias}\left(t\right)=\frac{1}{N_{t}}\sum_{i\in I_{t}}\left(y_{i}-{\hat{y}}_{i}\right),$$

where $I_{t}$ denotes the set of indices of data points corresponding to target label t. In addition, geometric LOR precision was evaluated on the point source dataset by measuring the median Euclidean distance between each produced LOR and the known point source location:

(9) $$d_{\mathrm{median}}=\mathrm{median}\left({\left\{d_{i}\right\}}_{i=1}^{N_{\text{LORs}}}\right),$$

where the use of the median reduces sensitivity to outliers arizing primarily from scattered events, present in the point source measurements but absent in the fan‐beam dataset. We also assessed the MAE on the fan‐beam data and $d_{\mathrm{median}}$ on the point source data under event‐filtering conditions. For the MAE analysis we generated an independent random train/validation/test split for each of the seven detectors in the middle ring, which provide complete data across all three spatial dimensions, and trained one model per detector. The final MAE was obtained by averaging the per‐detector test errors, with the associated standard deviation reflecting both data‐split stochasticity and intrinsic detector‐to‐detector variability. This detector‐level aggregation provides a conservative estimate of generalization performance as it evaluates the models across independently trained instances exposed to distinct detector characteristics.

Furthermore, reconstructed images $\hat{I}$ were analyzed with and without uncertainty‐aware filtering and weighting, using the two‐dimensional peak signal‐to‐noise ratio (PSNR), computed on the sum of all slices containing the rods. In addition, we computed the structural similarity index (SSIM) to quantify perceived structural fidelity. Given the ground‐truth image I, the PSNR and SSIM are defined as:

(10) $$\begin{array}{cc} \text{PSNR} & =10\cdot \log_{10}\left(\frac{\text{MAX}{\left(I\right)}^{2}}{\text{MSE}(I,\hat{I})}\right) \end{array}$$

(11) $$\begin{array}{cc} \text{SSIM} & =\frac{\left(2\mu_{I}\mu_{\hat{I}}+C_{1}\right)\left(2\sigma_{I\hat{I}}+C_{2}\right)}{\left(\mu_{I}^{2}+\mu_{\hat{I}}^{2}+C_{1}\right)\left(\sigma_{I}^{2}+\sigma_{\hat{I}}^{2}+C_{2}\right)} \end{array}$$

where $\mu_{I}$ and $\mu_{\hat{I}}$ represent mean intensities, $\sigma_{I}^{2}$ and $\sigma_{\hat{I}}^{2}$ are the variances, and $\sigma_{I\hat{I}}$ the covariance. To assess noise uniformity, we report the coefficient of variation (COV) within a uniformly filled phantom region. A binary mask Ω was derived from the ground‐truth phantom to select the uniform area, and the COV was computed as:

(12) $$\text{COV}=100\times \frac{\sigma_{\Omega }\left(\hat{I}\right)}{\mu_{\Omega }\left(\hat{I}\right)},$$

where $\mu_{\Omega }\left(\hat{I}\right)$ and $\sigma_{\Omega }\left(\hat{I}\right)$ denote the mean and standard deviation of the reconstructed activity within Ω, respectively (reported in %). In addition, line profiles were evaluated, and peak‐to‐valley ratios (PVR) were computed by averaging profile‐based PVR values for each rod diameter. Furthermore, we report rod‐wise activity recovery coefficients (RC) for all diameters of the hotrod phantom. For each rod, a GT‐informed local search identifies the reconstructed peak, and activity is averaged within a small neighborhood around it. Let ${\bar{A}}_{\mathrm{rod},d}$ denote the mean recovered activity for rods of diameter d, and ${\bar{A}}_{\mathrm{bg}}$ the mean background activity. The recovery coefficient is defined as

(13) $${\mathrm{RC}}_{d}=\frac{{\bar{A}}_{\mathrm{rod},d}}{{\bar{A}}_{\mathrm{bg}}}.$$

Final training of all calibration models, list‐mode processing, and image reconstruction were executed in a distributed manner across four NVIDIA RTX 6000 Ada Generation GPUs. Generative AI tools, including ChatGPT and Cursor, were used for limited code generation and language refinement; all outputs were critically reviewed, validated, and corrected by the authors.

## 3 RESULTS

### 3.1 Hyperparameter optimization

**FIGURE 2:**
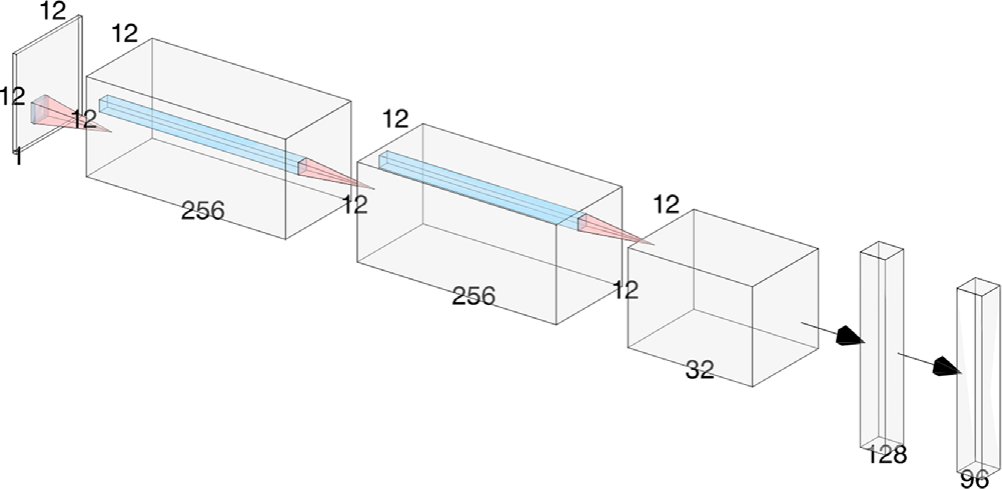
Optimized CNN classifier architecture identified through HPO for the monolithic prediction task. Convolutional kernel sizes are 5×5, 5×5, and 3×3. Each convolutional block comprises convolution, batch normalization, ReLU nonlinearity, and dropout. Final outputs are softmax‐normalized. The schematic was generated using the NN‐SVG tool by LeNail.^37^

HPO results are summarized in Tables 1, 2. The CNN classifier for the monolithic prediction task is visualized in Figure 2. All best‐performing models have parameter counts in the low‐million range. For MLPs, monolithic prediction models favor deeper architectures, in contrast to the segmented and DOI prediction tasks, which perform better with shallower configurations. This trend reverses for CNNs, where segmented and DOI models benefit from deeper structures. Overall, the optimal architectures differ substantially between segmented, monolithic, and DOI prediction tasks, indicating inherently different modelling requirements for each task. All subsequent results are based on the selected model configurations in Tables 1, 2.

**TABLE 1:** Optimized hyperparameter configurations for MLP‐based models across segmented, monolithic, and DOI tasks identified through Optuna‐based hyperparameter optimization.

Type	Model	Dropout	Layers	Layer sizes	LR	∼ Params
Reg.	Seg	0.0342	3	$2^{9}$, $2^{11}$, $2^{8}$	0.000415	$1.7\times 10^{6}$
	Mono	0.0113	5	$2^{9}$, $2^{9}$, $2^{9}$, $2^{9}$, $2^{8}$	0.000437	$1.0\times 10^{6}$
	DOI	0.00501	3	$2^{11}$, $2^{11}$, $2^{9}$	0.000557	$5.6\times 10^{6}$
Clf.	Seg	0.00503	3	$2^{10}$, $2^{9}$, $2^{9}$	0.000417	$1.0\times 10^{6}$
	Mono	0.0123	5	$2^{11}$, $2^{10}$, $2^{8}$, $2^{9}$, $2^{10}$	0.000555	$3.4\times 10^{6}$
	DOI	0.00699	4	$2^{10}$, $2^{8}$, $2^{11}$, $2^{7}$	0.000457	$1.2\times 10^{6}$

**TABLE 2:** Optimized hyperparameter configurations for CNN‐based models across segmented, monolithic, and DOI tasks identified through Optuna‐based hyperparameter optimization.

Type	Model	Dropout	Layers	Filters	Kernels	FC size	LR	∼ Params
Reg.	Seg.	0.0149	4	$2^{8}$, $2^{8}$, $2^{5}$, $2^{5}$	5, 5, 5, 5	256	0.00279	$3.1\times 10^{6}$
	Mono.	0.0209	2	$2^{8}$, $2^{7}$	5, 5	256	0.00204	$5.5\times 10^{6}$
	DOI	0.0237	4	$2^{8}$, $2^{8}$, $2^{8}$, $2^{6}$	3, 3, 3, 5	32	0.00342	$1.9\times 10^{6}$
Clf.	Seg.	0.00589	5	$2^{8}$, $2^{7}$, $2^{7}$, $2^{6}$, $2^{6}$	5, 5, 5, 5, 3	64	0.00112	$1.5\times 10^{6}$
	Mono.	0.00501	3	$2^{8}$, $2^{8}$, $2^{5}$	5, 3, 3	128	0.000453	$1.3\times 10^{6}$
	DOI	0.0236	4	$2^{8}$, $2^{7}$, $2^{8}$, $2^{3}$	3, 3, 3, 3	64	0.00100	$0.7\times 10^{6}$

### 3.2 MAE and bias distribution

**FIGURE 3:**
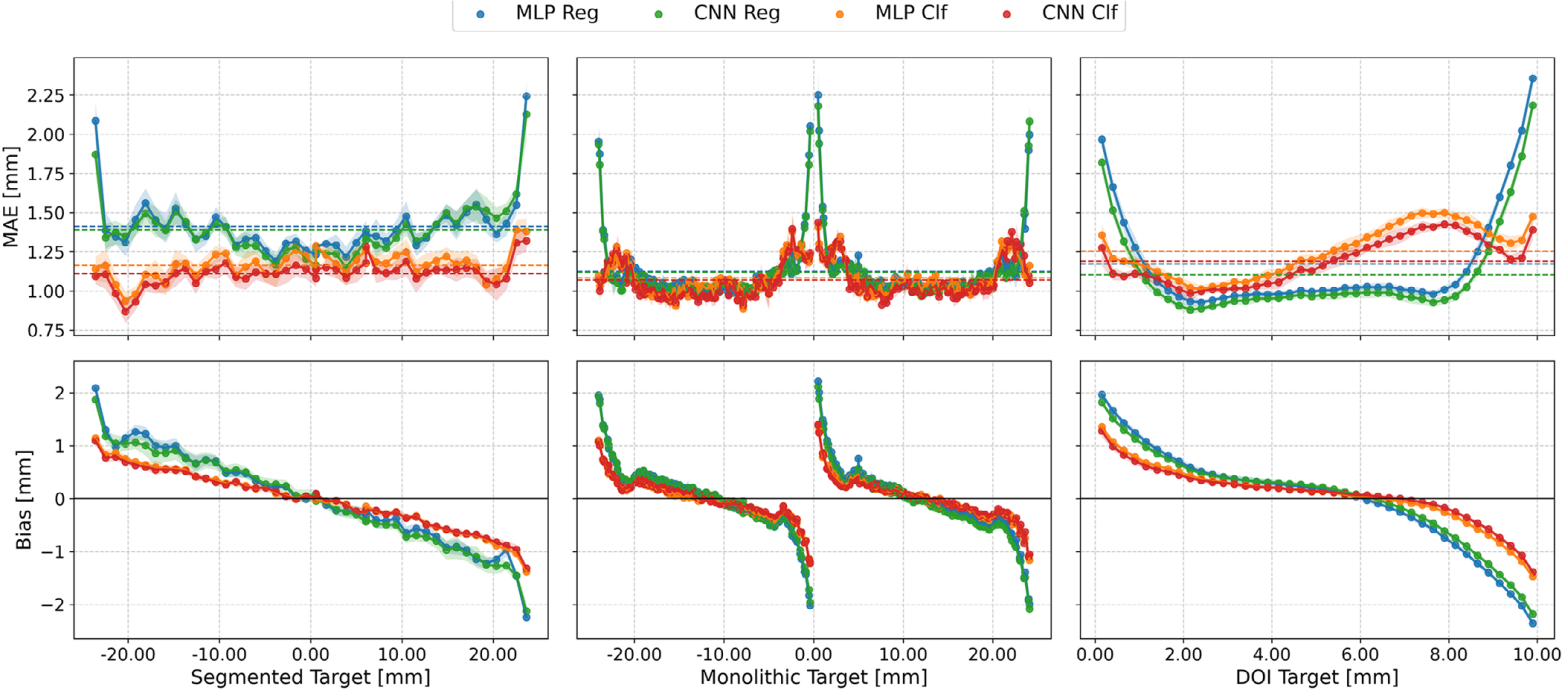
MAE and bias per target label for all model configurations evaluated on the seven detectors of the scanner's middle ring. No energy filtering is applied. Shaded regions denote the standard deviation across detectors, and horizontal lines indicate the model‐specific mean MAE across all target values.

**FIGURE 4:**
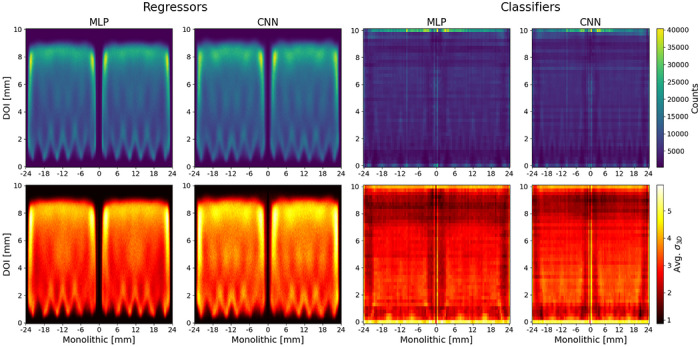
Monolithic‐DOI projections of flood irradiation (top) and averaged $\sigma_{\text{3D}}$ (bottom) across all detector modules for cylindrical shell phantom measurement. Regression outputs are histogrammed using 0.1mm bins, whereas classifier predictions are binned according to the discrete label resolution of the fan‐beam dataset: 0.5mm in the monolithic dimension and 0.25mm in DOI.

Figure 3 shows the MAE and bias as a function of target position across detectors for all model configurations. Pronounced edge effects are observed across all spatial dimensions. Along the segmented direction, the MAE increases markedly near the lateral detector boundaries, while in the monolithic direction elevated errors occur both at the outer crystal edges and at the interface between the two slab arrays. Similarly, in the DOI axis, the error increases within the uppermost and lowermost 2mm of the crystal. In the segmented and monolithic directions, an oscillatory pattern in the MAE is visible, which is consistent with the spatial discretization introduced by the SiPM readout grid. Across all target positions, classification models consistently exhibit lower bias magnitudes compared to regression models, with a notably different bias distribution in the DOI dimension. This behavior is also reflected in the flood maps obtained from the cylindrical shell measurement, visualized in Figure 4 (top). Regression models display regions of near‐zero predictions, whereas classification models provide predictions across the full detector extent. Comparing network architectures, CNNs consistently outperform MLPs in both MAE and bias characteristics. For DOI estimation, regression models achieve lower overall errors, with the CNN regressor yielding the best performance among all DOI models. Overall, the CNN classifier represents the most favorable trade‐off among the evaluated configurations, achieving the lowest planar MAE in both segmented and monolithic directions while maintaining the smallest bias magnitudes across the detector.

### 3.3 Variance, energy and MAE

Figure 4 (bottom) presents the average predicted $\sigma_{\text{3D}}$ per crystal voxel for all model configurations across a monolithic‐DOI projection. For regression models the highest variance values occur in the upper DOI layers as well as towards the edges of the slab arrays. For the classification models highest variances are predicted for the very top and bottom DOI layers, and slightly elevated toward the centers of the slab arrays. The elevated variance near the DOI boundaries is attributed to reduced light sharing near the photosensor, which limits DOI discrimination in the bottom DOI layers, and increased light spread and reflections near the opposite crystal surface, which increases overlap between DOI class light distributions.

**FIGURE 5:**
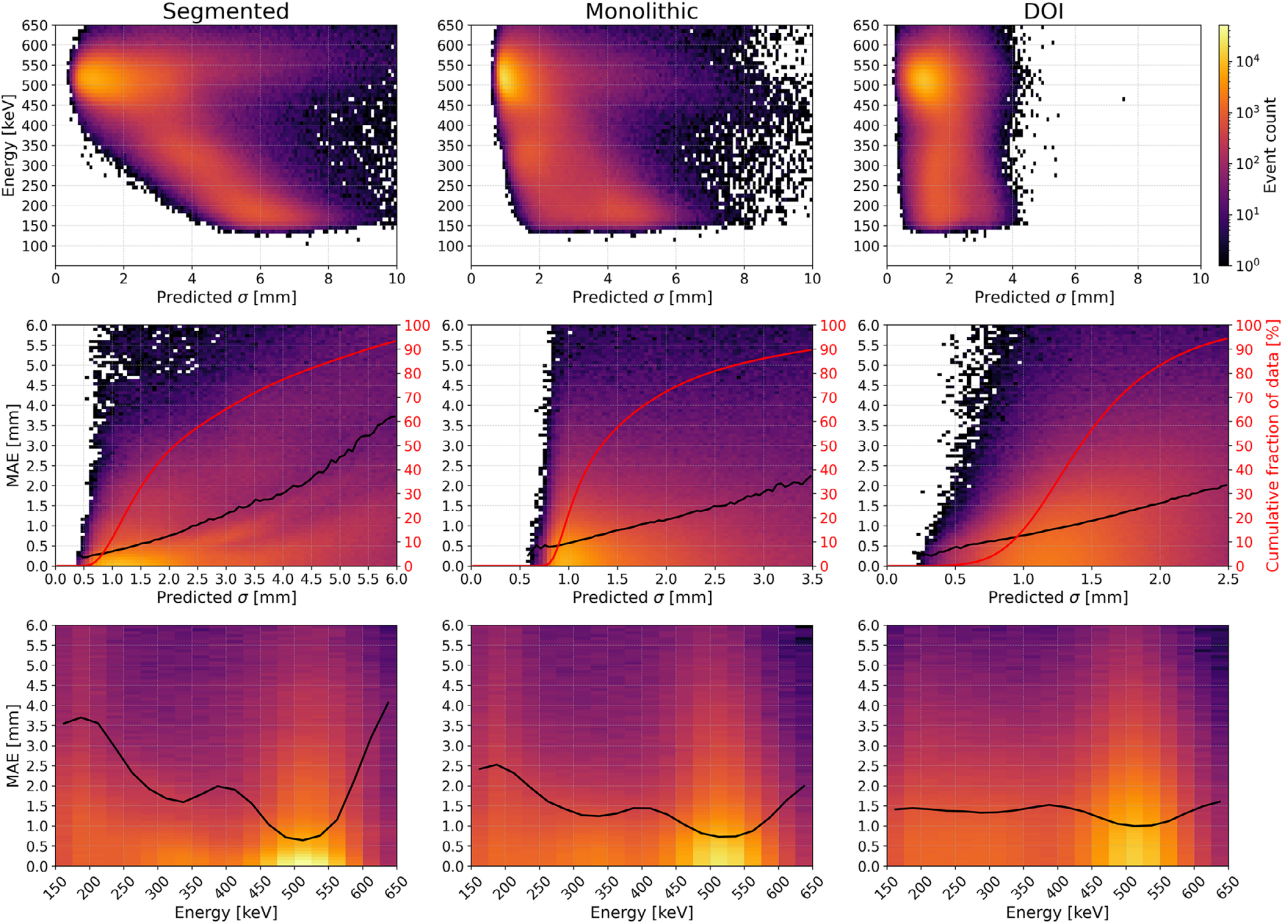
Performance results of the MLP regressor. (Top) Deposited energy as a function of predicted σ. (Middle) MAE as a function of predicted σ (black) with the cumulative fraction of predictions in red. (Bottom) MAE as a function of deposited energy with identical encoding.

Figure 5 (top) shows the relationship between deposited energy and the predicted $\sigma_{\text{3D}}$ of the MLP regressor. The distribution exhibits several characteristic structures across the segmented, monolithic, and DOI dimensions. The most prominent feature is the compact cluster of 511keV events with uniformly low predicted variance. These interactions provide maximal photon statistics and therefore yield the most precise spatial estimates across all coordinates. Beyond this full‐energy peak, the data reveal a broad inverse relationship between deposited energy and predicted variance in the planar dimensions: as deposited energy decreases, the model assigns increasing uncertainty. The reduced scintillation yield provides a plausible explanation for the diminished positional certainty. The relationship is not strictly monotonic, however. A distinct population of low‐energy events with low variance appears, most notably in the monolithic and DOI dimensions. These interactions would be rejected under an energy‐based filter but retained under a variance‐based criterion, suggesting that certain low‐light events can remain geometrically well constrained, possibly when occurring in regions of elevated intrinsic sensitivity such as directly above SiPMs. In addition, a band of moderately sub‐511keV energies accompanied by elevated variance is visible. Because a single Compton interaction cannot deposit these energies, this pattern likely reflects multiple‐scatter events, in which composite scintillation light distributions lead to increased localization uncertainty.

Figure 5 (middle) illustrates the relationship between predicted variance and MAE. Across all three spatial dimensions, a clear, monotonically increasing trend emerges: higher predicted variances are associated with increased MAEs. Additionally, the dispersion of the MAE increases with increasing variance, indicating a heavy‐tailed error distribution. Notably, the mean MAE remains below 1mm for predicted variances below approximately 2.5mm in the segmented direction, 1.75mm in the monolithic direction, and 1.5mm in the DOI dimension.

Figure 5 (bottom) illustrates the relationship between gamma photon energy and MAE. In contrast to the monotonic dependence observed with predicted $\sigma_{\text{3D}}$, the MAE as a function of energy exhibits a distinctly non‐monotonic structure. The lowest errors are found near 511keV, consistent with fully‐absorbed, unscattered photons. Predictions associated with energies exceeding 511keV, indicative of poor energy resolution or pile‐up effects and LYSO background noise, show substantially higher errors. A secondary minimum is observed around 340keV, corresponding to Compton backscatter events, where the photon undergoes a $180^{\circ }$ scatter, likely generating only a single light cluster. Between these two minima lies a region of elevated MAE, suggestive of multiple scattering events, as a single gamma photon cannot physically deposit such intermediate energy without undergoing successive interactions.

### 3.4 MAE, LOR precision and filtering

MAE performance results averaged across seven detectors under different filtering thresholds are summarized in Table 3. Filtering was applied only during evaluation, as all available data were used during training for both position and variance estimation. The corresponding standard deviations of the MAE values across detectors are reported in Table A1. The maximum observed inter‐detector deviation is 0.028mm. Unlike variance, the relationship between energy and MAE is non‐monotonic, which makes simple threshold‐based filtering suboptimal. To obtain a controlled fraction of retained events, we therefore define symmetric energy windows centered on the 511keV photopeak. A window of 511keV ± 80keV retains approximately 70% of the data, while 511keV ± 35keV retains roughly 50%.

**TABLE 3:** Fan‐beam mean MAE performance for all models under singles‐based filtering. For the energy‐ and variance‐only configurations, the reported percentages (100%, 70%, 50%) denote the fraction of singles retained after applying the corresponding threshold. For the variance‐after‐energy configuration, singles are first filtered using an energy window of 511 keV ± 80 keV (70% retention) and subsequently by variance‐based filtering; the reported percentages therefore refer to the fraction retained after the variance filter, resulting in an effective data retention of 70%×p (e.g., 49% for the 70% setting).

Filter	Model	Type	Segmented [mm]			Monolithic [mm]			DOI [mm]		
			100%	70%	50%	100%	70%	50%	100%	70%	50%
Energy	MLP	Regressor	1.412	0.830	0.688	1.126	0.821	0.752	1.171	1.065	1.015
		Classifier	1.163	0.644	0.514	1.085	0.795	0.726	1.254	1.108	1.052
	CNN	Regressor	1.390	0.806	0.669	1.118	0.811	0.741	1.102	1.011	0.969
		Classifier	1.111	0.611	0.488	1.073	0.787	0.720	1.189	1.071	1.027
Variance	MLP	Regressor	1.412	0.667	0.468	1.126	0.749	0.637	1.171	1.046	0.951
		Classifier	1.163	0.453	0.251	1.085	0.680	0.545	1.254	1.062	0.917
	CNN	Regressor	1.390	0.622	0.436	1.118	0.724	0.602	1.102	1.003	0.901
		Classifier	1.111	0.430	0.245	1.073	0.673	0.533	1.189	1.034	0.895
Variance after energy	MLP	Regressor	0.830	0.461	0.383	0.821	0.629	0.574	1.065	0.947	0.883
		Classifier	0.644	0.249	**0.171**	0.795	0.537	0.479	1.108	0.913	0.840
	CNN	Regressor	0.806	0.434	0.365	0.811	0.595	0.541	**1.011**	0.897	0.832
		Classifier	**0.611**	**0.243**	0.175	**0.787**	**0.527**	**0.470**	1.071	**0.892**	**0.827**

*Note*: Bold numbers indicate the best result per column.

**TABLE 4:** Median distance of LORs to reference point sources under filtering windows as defined in Table 3.

Model	Type	Energy [mm]			Variance [mm]			Energy+Variance [mm]		
		100%	70%	50%	100%	70%	50%	100%	70%	50%
MLP	Regressor	20.741	6.424	2.459	20.741	1.915	1.174	6.424	1.375	1.038
	Classifier	20.695	7.009	2.538	20.695	1.924	1.124	7.009	1.339	**0.983**
CNN	Regressor	20.688	**6.349**	**2.399**	20.688	**1.792**	**1.121**	**6.349**	**1.314**	0.990
	Classifier	**20.678**	6.876	2.460	**20.678**	1.960	1.152	6.876	1.357	0.993

*Note*: Bold numbers indicate the best result per column.

**TABLE 5:** PSNR, SSIM, and COV for reconstructed images obtained using MLP‐ and CNN‐based calibration models for regression and classification targets.

Model		PSNR	SSIM	COV
MLP	Reg.	14.550	0.403	5.35%
	Clf.	14.589	0.403	4.95%
CNN	Reg.	14.563	0.405	5.17%
	Clf.	**14.603**	**0.405**	**4.90%**

*Note*: Bold numbers indicate the best result per column.

**TABLE 6:** PVRs for reconstructed images across all rod diameters obtained using MLP‐ and CNN‐based calibration models for regression and classification targets.

Model		2.0 mm	1.5 mm	1.2 mm	1.0 mm	0.9 mm	0.8 mm
MLP	Reg.	3.8455	2.1345	1.5344	1.2458	1.1279	1.1011
	Clf.	4.1274	2.3473	1.6008	1.2766	1.1294	1.0989
CNN	Reg.	3.9602	2.1434	1.5461	1.2516	**1.1330**	**1.0989**
	Clf.	**4.1421**	**2.3628**	**1.6216**	**1.2974**	1.1317	1.0985

*Note*: Bold numbers indicate the best result per column.

**TABLE 7:** PSNR, SSIM, and COV results for reconstructed images obtained under different event‐filtering and count‐statistics conditions.

Singles variance filter	Used counts	PSNR	SSIM	COV
—	$250\cdot 10^{6}$	14.603	0.405	4.90%
70% (no sens. loss)	$250\cdot 10^{6}$	14.817	0.450	**4.63%**
50% (no sens. loss)	$250\cdot 10^{6}$	**14.899**	**0.462**	4.66%
70%	$125\cdot 10^{6}$	14.815	0.449	5.78%
50%	$62.5\cdot 10^{6}$	14.895	0.461	7.49%

*Note*: Bold numbers indicate the best result per column.

**TABLE 8:** PVRs for reconstructed images across all rod diameters obtained under different event‐filtering and count‐statistics conditions.

Configuration	2.0 mm	1.5 mm	1.2 mm	1.0 mm	0.9 mm	0.8 mm
250M	4.1421	2.3628	1.6216	1.2974	1.1317	1.0985
250M (70%)	6.4157	3.1980	1.9044	1.4366	1.1842	1.1526
250M (50%)	**7.4534**	**3.5770**	**2.0446**	**1.5089**	**1.2246**	**1.1840**
125M (70%)	6.3924	3.1962	1.9220	1.4426	1.1855	1.1536
62.5M (50%)	7.3201	3.5595	2.0339	1.5216	1.2224	1.1835

*Note*: Bold numbers indicate the best result per column.

**TABLE 9:** PSNR, SSIM, and COV for reconstructed images obtained under different LOR‐weighting configurations.

	**PSNR**	**SSIM**	**COV**
—	14.6029	0.4053	**4.9030%**
p=1	14.7066	0.4261	5.4280%
p=2	14.7923	0.4424	6.4196%
p=4	**14.8836**	**0.4620**	10.1874%

*Note*: Bold numbers indicate the best result per column.

**TABLE 10:** PVRs for reconstructed images across all rod diameters obtained under different LOR‐weighting configurations.

	2.0 mm	1.5 mm	1.2 mm	1.0 mm	0.9 mm	0.8 mm
—	4.1421	2.3628	1.6216	1.2974	1.1317	1.0985
p=1	5.0000	2.7417	1.7506	1.3425	1.1537	1.1334
p=2	5.8358	3.0700	1.8607	1.4067	1.1784	1.1623
p=4	**7.3905**	**3.4800**	**1.9975**	**1.5147**	**1.2233**	**1.2171**

*Note*: Bold numbers indicate the best result per column.

Without any filtering, the CNN classifier performs best in the planar positioning tasks and is slightly outperformed by the CNN regressor in DOI prediction. The CNN classifier achieves MAEs of approx. 1.1mm in all three spatial dimensions. Energy filtering to 50% of the data improves the MAEs down to approx. 0.49, 0.72 and 1.03mm. This is outperformed by the variance‐based filtering retaining the same amount of data, yielding improved MAEs of 0.25, 0.53, and 0.9mm, corresponding to near‐perfect label identification in planar positioning. As the fan‐beam dataset however does not contain any phantom scatter, in practice, both energy and variance filters have to be applied jointly. Further performance improvements can be observed, lowering the MAEs to 0.17, 0.47, and 0.83mm at the additional expense of again discarding approximately 50% of the data.

However, given a label precision of 0.5mm in the monolithic dimension and 0.25mm in DOI, together with a fan‐beam width of approximately 0.5mm, performance at or below this scale must be interpreted with caution. To further investigate geometric accuracy while evading these limitations, the median distance between reconstructed LORs and reference point source locations, was evaluated (cf. Table 4). Energy‐based filtering consistently reduces this distance, reflecting a suppression of phantom‐scattered events. Variance‐based filtering yields an even stronger improvement, indicating an implicit capturing of energy‐related information in the variance. The combination of energy and variance filtering achieves the best performance across all models, indicating a complementary effect and most effective suppression of mispositioned LORs, achieving upto sub‐mm distances.

These results show, that the segmented‐direction models show the strongest benefit from filtering, with a steep reduction of the MAE as high‐variance predictions are excluded. This, however, may reflect the inherently lower label resolution in the segmented dimension. In contrast, the monolithic and DOI models exhibit more modest improvements, with performance gains saturating quickly after minimal filtering. Across architectures, CNNs consistently outperform MLPs, and classifier‐based models generally surpass regressors, except for the DOI task. With combined energy‐ and variance‐based filtering applied, sub‐millimeter LOR precision can be achieved.

### 3.5 Image reconstruction

**FIGURE 6:**
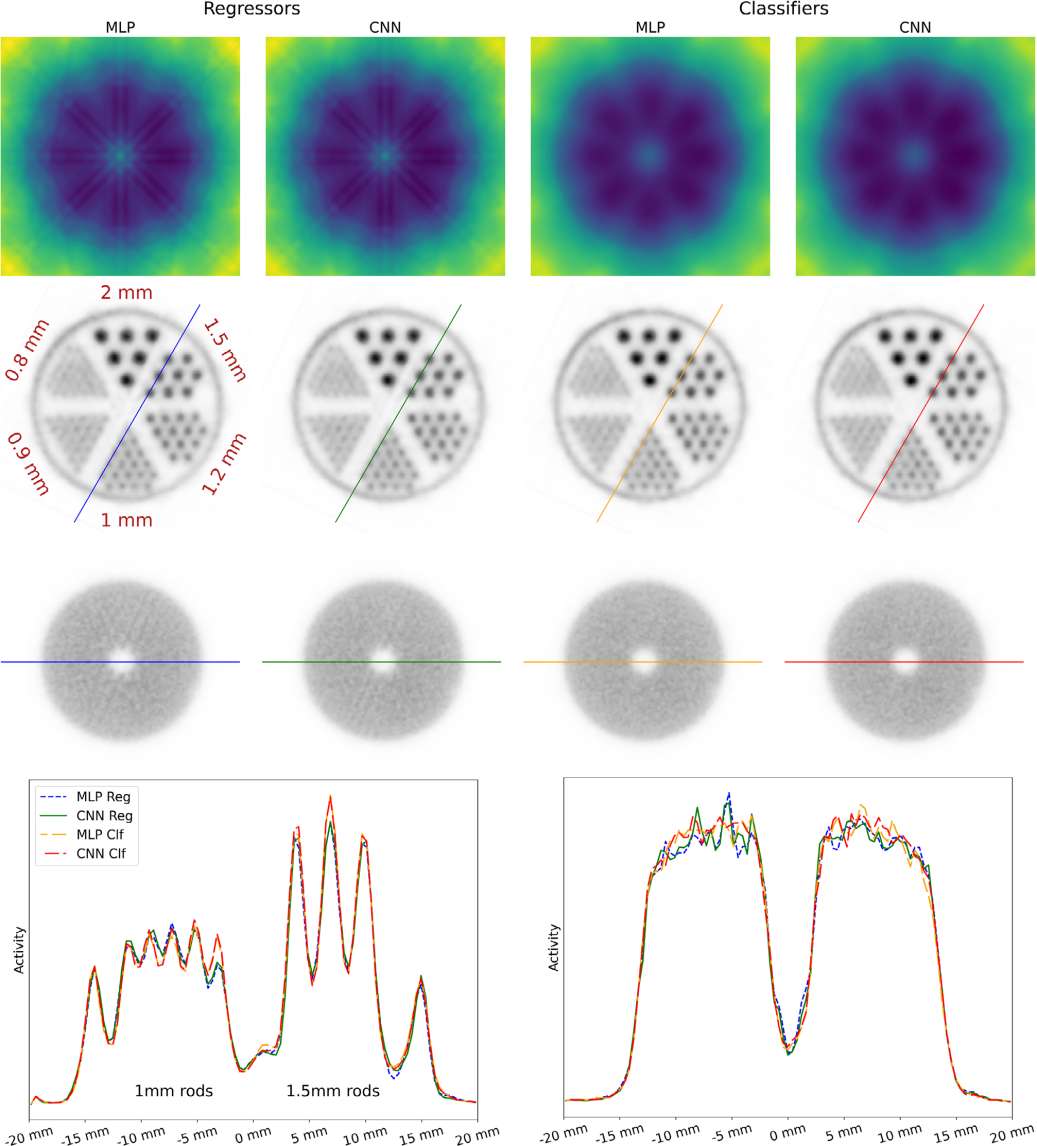
(Top) Sensitivity maps and reconstructed image slices for the rod and homogeneous region obtained using MLP‐ and CNN‐based calibration models for regression and classification targets. (Bottom) Corresponding line profile plots.

Images were reconstructed from list‐mode raw phantom data using the trained models. All reconstructions employed an energy window of 511keV ± 80keV. Figure 6 shows the reconstructed images for the different model configurations. Visual inspection clearly shows the classifiers outperforming the regressors, with the regressor images showing higher background noise and stripe artefacts oriented toward the direction of the slab‐array edges, which can also be seen in the sensitivity maps. Line profiles confirm this trend. PSNR, SSIM, and COV results, given in Table 5, further validate this, showing the CNN classifier outperforming the other configurations in all metrics and achieving a coefficient of variation of 4.9%. In terms of PVRs, given in Table 6, the CNN classifiers also perform best, except for the smallest rods where the CNN regressor shows a slight advantage.

**FIGURE 7:**
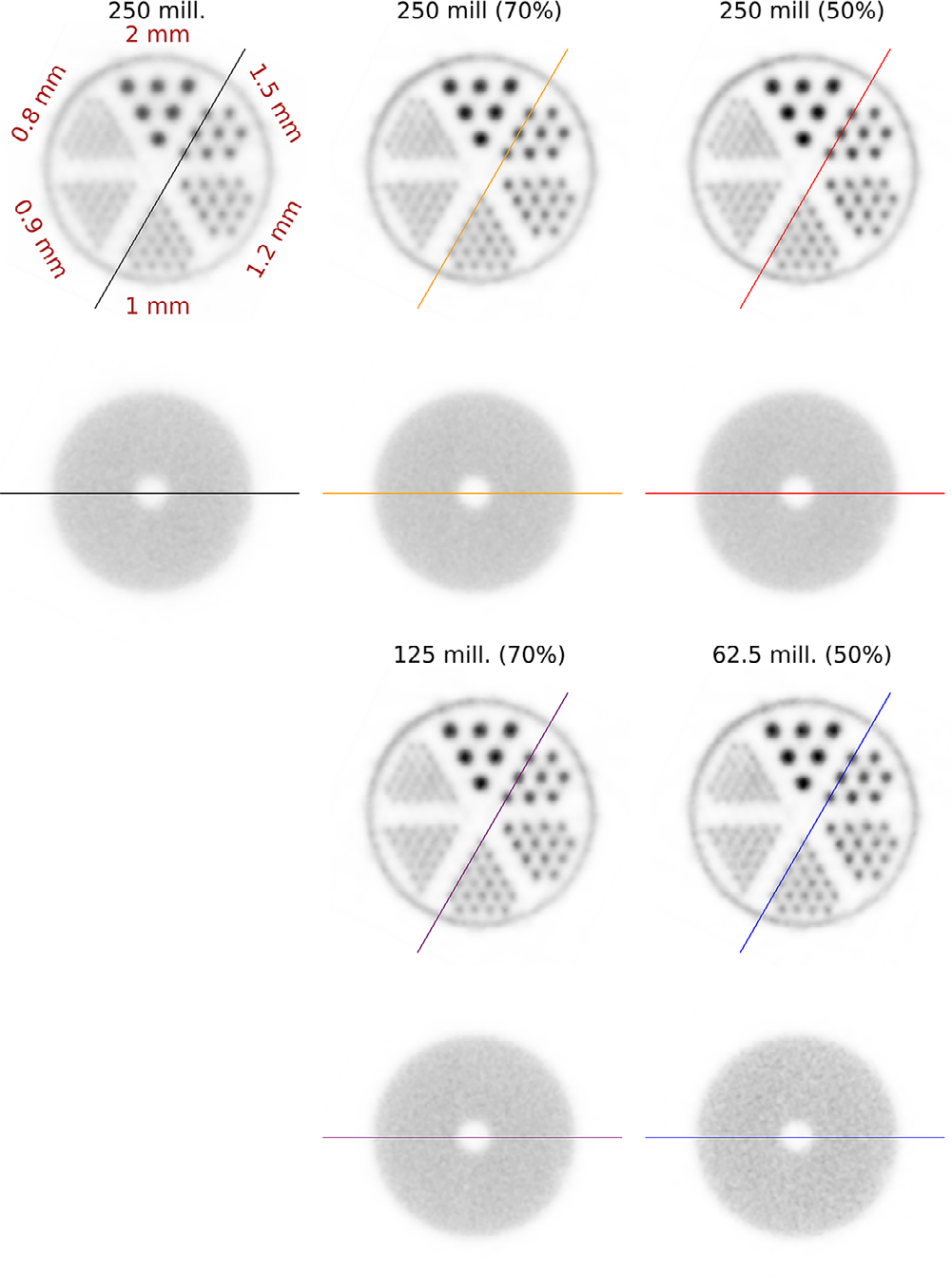
Reconstructed image slices for the rod and homogeneous regions obtained using 70% and 50% singles retention filters, with and without the corresponding loss of sensitivity. The percentage and the number shown above each image indicate the singles retention level and the number of used coincidence events after filtering, respectively.

**FIGURE 8:**
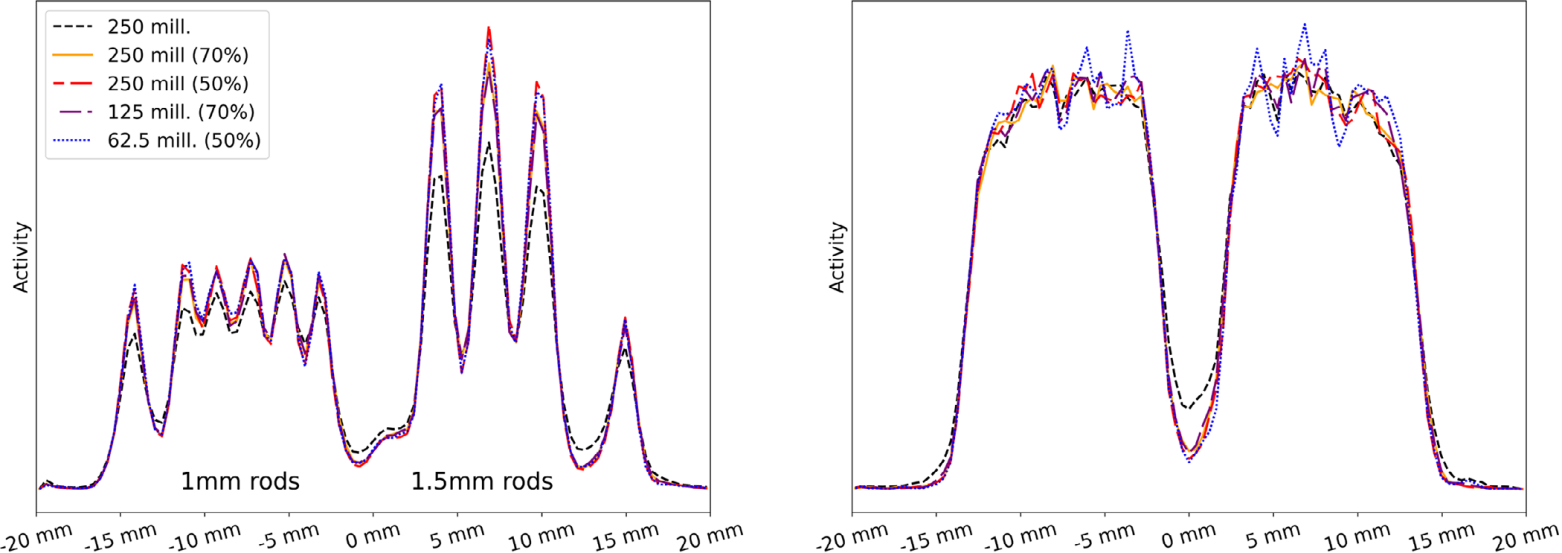
Line profile plots obtained using 70% and 50% singles retention filters, with and without the corresponding loss of sensitivity.

We further applied the trained CNN classification models to reconstruct images using variance‐filtered data, both with and after restoring the effective statistics to match the unfiltered number of coincidences. Filter parameters were derived from the cylindrical shell phantom measurement. For each filter, we recomputed the normalization and performed the corresponding image reconstruction. Images and line profiles are shown in Figures 7, 8. Table 7 reports the PSNR, SSIM, and COV values for the different filters. It can be observed that the PSNR increases with a stronger variance filter applied to the coincidences. When statistics are restored to the unfiltered coincidence count, all metrics improve, whereas when reconstruction is performed using the reduced coincidence statistics after filtering, PSNR and SSIM improve while the COV degrades. As this behavior is not observed when using the full coincidence statistics, we attribute it to increased Poisson noise resulting from the reduced event count. PVRs, given in Table 8, improve across all rod diameters when comparing the original dataset to the filtered data without restoring the statistics. The rod‐wise RCs (Figure 10) are higher for all filtering conditions than for the unfiltered baseline, with the highest RCs obtained for the full‐statistics reconstruction at 50% singles filtering.

**FIGURE 9:**
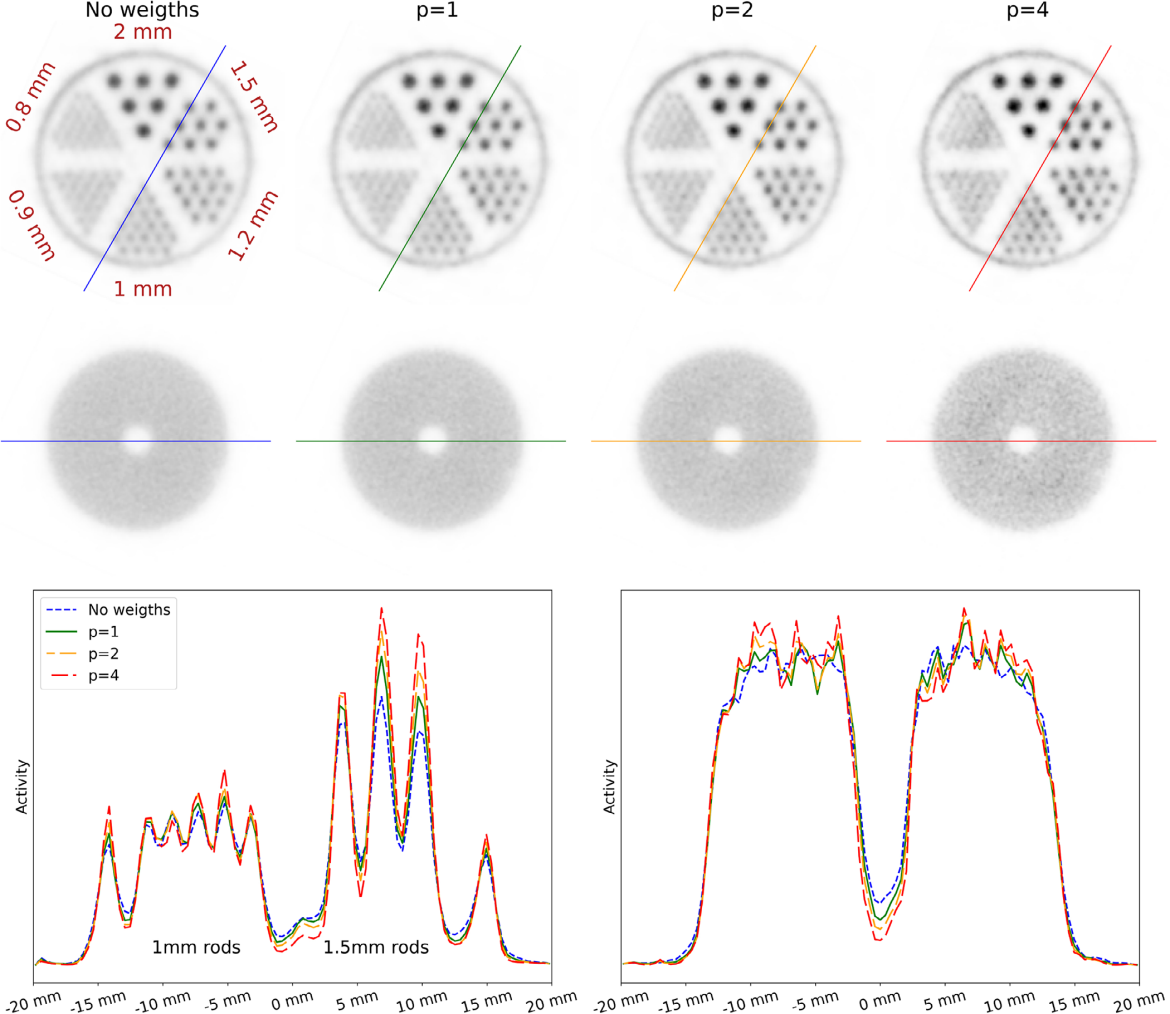
(Top) Reconstructed image slices of the rod and homogeneous regions obtained using LOR weighting. (Bottom) Corresponding line profile plots.

**FIGURE 10:**
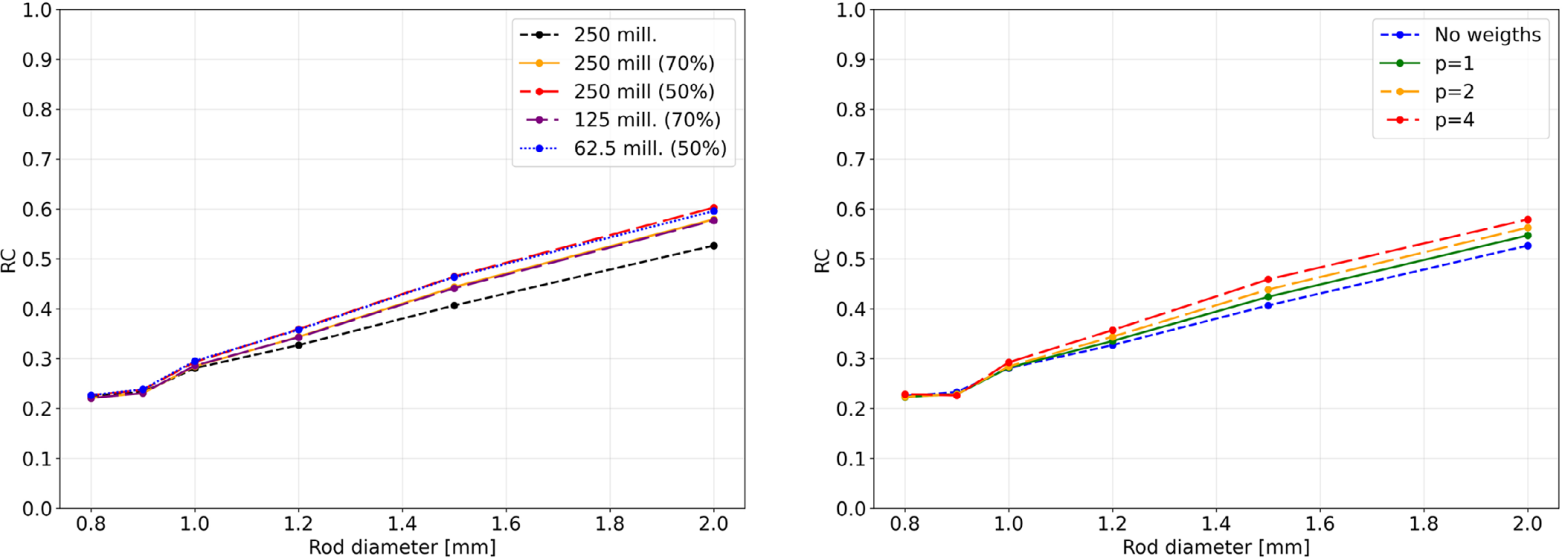
Rod‐wise activity recovery coefficients as a function of rod diameter for event‐level filtering (left) and LOR weighting (right).

Lastly, we reconstructed images by weighting LORs by the uncertainty metric $w_{\text{LOR}}$, visualized in Figure 9, and quantified in Tables 9, 10. PSNR, SSIM, and PVR values show clear improvements with increasing weighting exponent, while the COV exhibits a pronounced degradation for p>2. However, as for event‐level filtering, the weighting approach consistently and monotonously improves rod‐wise RCs, yielding the highest RC for p=4 (cf. Figure 10).

## 4 DISCUSSION

Our results demonstrate that optimizing gamma interaction localization via uncertainty‐aware distribution matching improves interaction positioning performance and enables a seamless integration into the PET data processing pipeline via filtering and weighting strategies. Image quality, as quantified by PSNR, SSIM, PVR, and RC can be improved even when accounting for reduced effective scanner sensitivity, while the COV increases due to elevated Poisson noise.

For the same detector topology, Kuhl et al. ^4^ achieved MAEs of 0.75mm in the planar‐monolithic direction and 1.03mm for DOI, which is comparable to our results of 0.79mm and 1.01mm obtained under near‐identical energy filtering. Although variance‐based filtering yields further reductions in the MAE, absolute improvements at this scale are difficult to interpret due to the limited label precision (0.5mm planar–monolithic, 0.25mm DOI) and the finite fan‐beam width of approximately 0.5mm. However, the complementary LOR‐to‐point‐source distance analysis provides a robust indication that both filtering strategies suppress mispositioned events, yielding median LOR‐to‐point‐source distances down to 0.983mm, approaching the physical resolution limits of the system, considering effects such as positron range, non‐collinearity, and misalignments of the detectors themselves, as analyzed by [27]. With proper calibration and uncertainty‐aware reconstruction algorithms in place, sub‐millimeter spatial image resolution can be achieved. Nevertheless, several challenges remain that warrant further investigation.

One key limitation of the present approach lies in the assumption of a Gaussian error model, embedded in both the loss function and uncertainty parametrization. Deviations are particularly evident for events involving Compton scatter, which produce large residuals and contribute to the heavy‐tailed error distribution that is not well described by a Gaussian likelihood. In addition, boundary effects are not explicitly encoded in the current loss formulation. As demonstrated by Daniel et al.,^25^ truncated Gaussian likelihoods more accurately reflect the physical constraints imposed by the detector boundaries. Depending on the predicted coordinate, folded or otherwise modified Gaussian forms may be more appropriate to account for light reflections and the limited support near crystal boundaries. Furthermore, incorporating richer representations of event‐level uncertainty into the reconstruction pipeline may offer further improvements. Instead of relying solely on predicted variances, one could leverage the full event‐wise predictive distribution, for example by sampling interaction positions according to the model's classification‐derived spatial probability maps during image reconstruction. This would provide a more faithful propagation of uncertainty and may further mitigate the influence of outlier events on the final image. However, such a sampling‐based approach is not optimal with respect to geometric precision. Because the distance between a LOR and an annihilation position is a convex function of the gamma interaction positions, Jensen's inequality^38^ implies that using the mean interaction position minimizes the expected LOR–annihilation distance. Sampling from the predictive distribution necessarily incurs a positive Jensen gap, increasing this expectation even for a perfectly calibrated model. This reflects a fundamental trade‐off between minimizing geometric bias and accurately propagating event‐dependent localization uncertainty into the reconstruction process via sampling.

The proposed uncertainty‐aware positioning approach is applicable to any detector concept employing light sharing, including the presented semi‐monolithic design, prism‐based detectors,^39^ detectors with light guides on the opposing side,^40^ and multi‐layer staggered crystal arrays.^27^ These detector designs encode both interaction position and event‐wise positioning ambiguity in the spatial distribution of scintillation light across multiple photosensors, making event‐wise uncertainty estimation directly relevant for positioning, event selection, and analysis of detector‐specific positioning limitations. By contrast, fully segmented detectors without substantial light sharing mainly provide crystal identification and therefore little continuous information about event‐specific positioning uncertainty. Light sharing also introduces a trade‐off between positioning precision and statistical noise: large amounts of light sharing improve geometric positioning but distribute photons over more channels, increasing relative Poisson noise per channel. The resulting positioning ambiguity can be captured within the uncertainty estimation framework. Compton scatter within the crystal can produce multiple light clusters, for which the detected light distribution no longer uniquely identifies the first interaction point. Because scintillation light emission is isotropic, the interaction sequence cannot be inferred from the light pattern alone, leading to intrinsically ambiguous positioning. Although uncertainty‐aware positioning models cannot resolve the first interaction in these cases, they can reflect the ambiguity through increased predicted variance, allowing Compton events to be identified and down‐weighted during reconstruction rather than mispositioned. Future work will analyze multi‐cluster events to relate light spread structure to predicted uncertainty and to characterize the impact of inter‐crystal Compton scatter on positioning uncertainty.

A related conceptual limitation concerns the interpretation of the predicted variances. In the present formulation, the network outputs a heteroscedastic variance term, which is typically associated with aleatoric uncertainty. However, because the variance is inferred through a deterministic model, its estimates may be affected by epistemic limitations such as finite training data or model capacity. The current framework does not disentangle these influences. More principled uncertainty modeling could therefore improve both calibration accuracy and downstream reconstruction performance. Jointly accounting for both epistemic and aleatoric sources of uncertainty could yield more reliable event‐level confidence estimates and enable more robust PET image reconstruction.

Another open issue remains the trade‐off between quantitative recovery and noise. In our study, uncertainty‐informed filtering and weighting increase rod‐wise RCs while, at the same time, raising uniform‐region COV due to reduced effective sensitivity. By discarding or down‐weighting events with high predicted uncertainty, spatial consistency and recovery improve, but fewer effective counts remain, which amplifies Poisson noise and can reduce quantitative precision in homogeneous regions. Addressing this limitation requires reconstruction strategies that preserve the benefits of uncertainty‐informed event selection while mitigating the resulting sensitivity loss. Ultimately, further progress in quantitative PET will require careful balancing of event selection, uncertainty modelling, and reconstruction physics.

Future work should also include ablation studies targeting more compact network architectures suitable for integration into FPGA‐based PET readout systems. Techniques such as network quantization and pruning could substantially reduce memory requirements and computational complexity, enabling real‐time deployment while maintaining predictive performance.

## 5 CONCLUSION

This work demonstrates that aleatoric uncertainty in gamma‐interaction positioning can be estimated using modern deep learning approaches and meaningfully integrated into the PET reconstruction pipeline. Through extensive hyperparameter optimization, we show that CNNs trained with a classification objective provide the strongest planar localization performance, while CNN‐based regressors achieve superior DOI precision. Across all evaluated architectures, event‐level uncertainty estimates can be obtained and exploited for variance‐based event filtering and uncertainty‐informed LOR weighting during reconstruction. Both strategies selectively suppress or downweight poorly localized interactions, leading to measurable improvements in the reconstructed image quality. These gains are accompanied by a trade‐off, as filtering and weighting reduce the effective scanner sensitivity and increase image noise, with potential implications for quantitative accuracy. Nevertheless, our results establish uncertainty‐informed event selection and weighting as principled extensions to PET data processing. Looking forward, further improvements are expected from richer uncertainty models beyond Gaussian assumptions and tighter coupling between uncertainty estimation and reconstruction algorithms, with more hardware‐efficient network designs enabling efficient and real‐time inference. Overall, uncertainty‐aware event modelling provides a concrete and extensible pathway toward higher‐resolution PET systems, with the potential to improve quantitative fidelity and increase confidence in downstream clinical interpretation.

## CONFLICT OF INTEREST STATEMENT

The authors declare no conflicts of interest.

## APPENDIX A

**TABLE A1:** Standard deviation of MAE across detectors for fan‐beam measurements obtained under different event‐filtering configurations and model types. Percentages follow the same interpretation as in Table 3.

Filter	Model	Type	Segmented [mm]			Monolithic [mm]			DOI [mm]		
			100%	70%	50%	100%	70%	50%	100%	70%	50%
Energy	MLP	Regressor	0.028	0.015	0.013	0.012	0.006	0.007	0.015	0.013	0.015
		Classifier	0.015	0.003	0.005	0.007	**0.004**	0.006	0.014	0.010	0.012
	CNN	Regressor	0.022	0.011	0.015	0.011	0.004	0.006	0.010	0.010	0.012
		Classifier	0.012	0.004	0.009	0.010	0.004	0.008	0.011	0.009	0.013
Variance	MLP	Regressor	0.028	0.024	0.017	0.012	0.007	0.006	0.015	0.012	0.013
		Classifier	0.015	0.007	0.007	0.007	0.004	0.006	0.014	0.010	0.010
	CNN	Regressor	0.022	0.017	0.018	0.011	0.004	0.007	0.010	0.009	0.008
		Classifier	0.012	0.006	**0.004**	0.010	0.005	0.006	0.011	0.011	0.009
Variance after energy	MLP	Regressor	0.015	0.017	0.019	0.006	0.006	0.006	0.013	0.013	0.012
		Classifier	**0.003**	0.007	0.006	**0.004**	0.006	**0.005**	0.010	0.010	0.009
	CNN	Regressor	0.011	0.017	0.018	0.004	0.007	0.008	0.010	**0.008**	**0.008**
		Classifier	0.004	**0.003**	0.004	0.004	0.005	0.007	**0.009**	0.009	0.008

*Note*: Bold numbers indicate the best result per column.

## References

[1] Wahl RL , Jacene H , Kasamon Y , Lodge MA . From recist to percist: evolving considerations for pet response criteria in solid tumors. J Nucl Med. 2009;50(S1):122S‐150S. 19403881 10.2967/jnumed.108.057307 PMC2755245

[2] Bal H , Guerin L , Casey M , et al. Improving pet spatial resolution and detectability for prostate cancer imaging. Phys Med Biol. 2014;59(15):4411. 25049221 10.1088/0031-9155/59/15/4411 PMC8375570

[3] van der Vos CS , Koopman D , Rijnsdorp S , et al. Quantification, improvement, and harmonization of small lesion detection with state‐of‐the‐art PET. Eur J Nucl Med Mol Imaging. 2017;44(S1):4‐16. 28687866 10.1007/s00259-017-3727-z PMC5541089

[4] Kuhl Y , Mueller F , Naunheim S , et al. A finely segmented semi‐monolithic detector tailored for high‐resolution PET. Med Phys. 2024;51(5):3421‐3436. 38214395 10.1002/mp.16928

[5] Zhang C , Sang Z , Wang X , Zhang X , Yang Y . The effects of inter‐crystal scattering events on the performance of PET detectors. Phys Med Biol. 2019;64(20):205004. 31530747 10.1088/1361-6560/ab44f4

[6] Galindo‐Tellez A , Sharyy V , Sung CH , et al. First clearmind gamma detector prototype for TOF‐PET imaging. J Instrum. 2024;19(07):P07037.

[7] Roncali E , Mosleh‐Shirazi MA , Badano A . Modelling the transport of optical photons in scintillation detectors for diagnostic and radiotherapy imaging. Phys Med Biol. 2017;62(20):R207. 28976914 10.1088/1361-6560/aa8b31 PMC5739055

[8] Yang X , Downie E , Farrell T , Peng H . Study of light transport inside scintillation crystals for PET detectors. Phys Med Biol. 2013;58(7):2143. 23470488 10.1088/0031-9155/58/7/2143

[9] Acerbi F , Gundacker S . Understanding and simulating SiPMs. Nucl Instrum Methods Phys Res, Sect A. 2019;926:16‐35.

[10] Li X , Lockhart C , Lewellen TK , Miyaoka RS . Study of PET detector performance with varying SiPM parameters and readout schemes. IEEE Trans Nucl Sci. 2011;58(3):590‐596. 22685348 10.1109/TNS.2011.2119378 PMC3368805

[11] Mueller F , Schug D , Hallen P , Grahe J , Schulz V . Gradient tree boosting‐based positioning method for monolithic scintillator crystals in positron emission tomography. IEEE Trans Radiat Plasma Med Sci. 2018;2(5):411‐421.

[12] Mueller F , Schug D , Hallen P , Grahe J , Schulz V . A novel DOI positioning algorithm for monolithic scintillator crystals in PET based on gradient tree boosting. IEEE Trans Radiat Plasma Med Sci. 2018;3(4):465‐474.

[13] Krueger K , Mueller F , Gebhardt P , Weissler B , Schug D , Schulz V . High‐throughput FPGA‐based inference of gradient tree boosting models for position estimation in pet detectors. IEEE Trans Radiat Plasma Med Sci 2023;7(3):253‐262.

[14] Carra P , Bisogni MG , Ciarrocchi E , et al. A neural network‐based algorithm for simultaneous event positioning and timestamping in monolithic scintillators. Phys Med Biol. 2022;67(13):135001. 10.1088/1361-6560/ac72f2 35609583

[15] Correia PMM , Cruzeiro B , Dias J , et al. Precise positioning of gamma ray interactions in multiplexed pixelated scintillators using artificial neural networks. Biomed Phys Eng Express. 2024;10(4):045038. doi:10.1088/2057-1976/ad4f73 38779912

[16] Jaliparthi G , Martone PF , Stolin AV , Raylman RR . Deep residual‐convolutional neural networks for event positioning in a monolithic annular PET scanner. Phys Med Biol. 2021;66(14):145008. 10.1088/1361-6560/ac0d0c PMC8908313 34153950

[17] Kawula M , Binder T , Liprandi S , Viegas R , Parodi K , Thirolf PG . Sub‐millimeter precise photon interaction position determination in large monolithic scintillators via convolutional neural network algorithms. Phys Med Biol. 2021;66(13):135017. 10.1088/1361-6560/ac06e2 34062523

[18] Sanaat A , Zaidi H . Accurate estimation of depth of interaction in pet on monolithic crystal coupled to SiPMs using a deep neural network and Monte Carlo simulations. In 2019 IEEE nuclear science symposium and medical imaging conference (NSS/MIC) , IEEE; 2019:1‐3.

[19] Decuyper M , Stockhoff M , Vandenberghe S , Van Holen R . Artificial neural networks for positioning of gamma interactions in monolithic PET detectors. Phys Med Biol. 2021;66(7):075001. 10.1088/1361-6560/abebfc 33662940

[20] Freire M , Barrio J , Cucarella N , et al. Position estimation using neural networks in semi‐monolithic pet detectors. Phys Med Biol. 2022;67(24):245011. 10.1088/1361-6560/aca389 36384047

[21] Lee S , Lee JS . Inter‐crystal scattering recovery of light‐sharing pet detectors using convolutional neural networks. Phys Med Biol. 2021;66(18):185004. doi:10.1088/1361-6560/ac215d 34438380

[22] Gross‐Weege N , Schug D , Hallen P , Schulz V . Maximum likelihood positioning algorithm for high‐resolution pet scanners. Med Phys. 2016;43(6Part1):3049‐3061. doi:https://aapm.onlinelibrary.wiley.com/doi/abs/10.1118/1.4950719 27277052 10.1118/1.4950719

[23] Lerche CW , Salomon A , Goldschmidt B , Lodomez S , Weissler B , Solf T . Maximum likelihood positioning and energy correction for scintillation detectors. Phys Med Biol. 2016;61(4):1650. 26836394 10.1088/0031-9155/61/4/1650

[24] Lerche CW , Bi W , Schoeneck M , et al. Fast maximum likelihood positioning for a staggered layer scintillation PET detector. arXiv:2503.13723. 2025. 10.1088/1361-6560/adf8ab 40769190

[25] Daniel G , Yahiaoui MB , Comtat C , et al. Deep learning reconstruction with uncertainty estimation for gamma photon interaction in fast scintillator detectors. Eng Appl Artif Intell. 2024;131:107876. https://www.sciencedirect.com/science/article/pii/S0952197624000344

[26] Akiba T , Sano S , Yanase T , Ohta T , Koyama M . Optuna: A next‐generation hyperparameter optimization framework. In Proceedings of the 25th ACM SIGKDD International Conference on Knowledge Discovery & Data Mining . 2019:2623‐2631.

[27] Thull J , Kuhl Y , Mueller F , Schug D , Weissler B , Schulz V . Beyond mechanics: maximum‐likelihood‐driven PET detector alignment calibration. Med Phys. 2026;53(1):e70256. 41474022 10.1002/mp.70256 PMC12754747

[28] Frach T , Prescher G , Degenhardt C , De Gruyter R , Schmitz A , Ballizany R . The digital silicon photomultiplier–principle of operation and intrinsic detector performance. In 2009 IEEE Nuclear Science Symposium Conference Record (NSS/MIC) , IEEE; 2009:1959‐1965.

[29] Hyperion Hybrid Imaging Systems . PET detector platform. https://hyperion‐his.com/pet‐detector‐platform/ 2025, accessed: 2025‐05‐26.

[30] Bovelett M , Kuhl Y , Naunheim S , Schug D , Schulz V , Mueller F . Implementation and evaluation of a 3D‐dependent energy‐calibration algorithm, including interpolation methods for missing read‐out channels. In 2023 IEEE Nuclear Science Symposium, Medical Imaging Conference and International Symposium on Room‐Temperature Semiconductor Detectors (NSS MIC RTSD) , IEEE; 2023:1‐1.

[31] Weissler B , Schug D , Dey T , et al. Hyperion III–a flexible pet detector platform for simultaneous PET/MRI. Nuklearmedizin. 2020;59(02):V93.

[32] Kuhl Y , Mueller F , Thull J , Naunheim S , Schug D , Schulz V . 3D in‐system calibration method for pet detectors. Med Phys. 2025;52(1):232‐245. 39504412 10.1002/mp.17475 PMC11699997

[33] Watanabe S . Tree‐structured parzen estimator: understanding its algorithm components and their roles for better empirical performance. arXiv:2304.11127. 2023.

[34] Paszke A , Gross S , Massa F , et al. Pytorch: an imperative style, high‐performance deep learning library. Adv Neural Inf Process Syst. 2019;32:8024‐8035.

[35] Shepp LA , Vardi Y . Maximum likelihood reconstruction for emission tomography. IEEE Trans Med Imaging. 2007;1(2):113‐122. 10.1109/TMI.1982.4307558 18238264

[36] Theodorakis L , Loudos G , Prassopoulos V , Kappas C , Tsougos I , Georgoulias P . A review of pet normalization: striving for count rate uniformity. Nucl Med Commun. 2013;34(11):1033‐1045. 24048410 10.1097/MNM.0b013e328365ac1e

[37] LeNail A . NN‐SVG: publication‐ready neural network architecture schematics. J Open Source Software. 2019;4(33):747. doi:10.21105/joss.00747

[38] Jensen JLWV . Sur les fonctions convexes et les inégalités entre les valeurs moyennes. Acta Math. 1906;30(1):175‐193.

[39] LaBella A , Cao X , Petersen E , et al. High‐resolution depth‐encoding pet detector module with prismatoid light‐guide array. J Nucl Med. 2020;61(10):1528‐1533. 32111684 10.2967/jnumed.119.239343 PMC7539654

[40] Terragni G , Nadig V , Tribbia E , et al. Exploring the performance of a DOI‐capable TOF‐PET module using different SiPMs, customized and commercial readout electronics. Phys Med Biol. 2025;70(2):025015. 10.1088/1361-6560/ada19a 39700624

